# Measures of adiposity correlate with renal filtration in young nulliparous women: An observational cohort study

**DOI:** 10.1002/osp4.375

**Published:** 2019-12-17

**Authors:** Frank B. Williams, Carole A. McBride, Gary J. Badger, Ira M. Bernstein

**Affiliations:** ^1^ Department of Obstetrics, Gynecology and Reproductive Services, Larner College of Medicine University of Vermont Burlington Vermont

## Abstract

**Objective:**

Renal hyperfiltration, which has been documented in severe obesity and obesity‐associated hypertension, can occur with hypertensive disorders of pregnancy. Identification of prepregnancy risk factors for unrecognized renal hyperfiltration could inform screening and intervention strategies to protect against pregnancy complications. In young, healthy, nulliparous women, associations between associations between measures of adiposity, insulin resistance, and renal vascular resistance were thus evaluated.

**Methods:**

This is a secondary analysis of a prospective observational trial characterizing associations of prepregnancy and late‐pregnancy maternal physiology. Seventy‐nine nulligravid women aged 18‐42 years without major medical conditions were assessed for percent android body fat using dual‐energy X‐ray absorption. Renal cortical vessel blood flow resistance index (CVRI) was determined using Doppler ultrasonography. Creatinine clearance was calculated from 24‐hour urine collection.

**Results:**

Renal CVRI inversely correlates with body mass index (r = ‐0.23, *p* = 0.047), percent android fat (r = ‐0.30, *p* = 0.008), and supine pulse (r = ‐0.44, *p* < 0.001). Creatinine clearance is positively associated with BMI and HOMA‐IR.

In regression modeling, supine pulse (r^2^ = 0.22, *p* < 0.001) and cardiac index (r^2^ = 0.05, *p* = 0.045) predict renal CVRI, whereas HOMA‐IR (r^2^ = 0.11, *p* = 0.008) and cardiac output (r^2^ = 0.06, *p* = 0.039) predict creatinine clearance. Measures of adiposity are not independently predictive of either measure.

**Conclusions:**

In healthy young women, measures of adiposity and insulin resistance correlate positively with renal filtration. Preclinical manifestations of renal hyperfiltration may have implications for pregnancy outcomes.

**Abbreviations**
WHO: World Health Organization
BMI: body mass index
MHz: megahertz
DEXA: dual‐energy X‐ray absorptiometry
HOMA‐IR: homeostatic model assessment of insulin resistance

## 1 INTRODUCTION

The majority of reproductive‐aged women in the United States are either overweight or obese as defined by body mass index (BMI) greater than 25.0 kg/m^2^.^1^ Conditions associated with obesity, including hypertension and metabolic syndrome, have been linked to elevated renal filtration. Obesity‐associated hypertension has been correlated with increased renal plasma blood flow.^2^, ^3^, ^4^, ^5^ Metabolic syndrome, as defined by central obesity (waist circumference of 88 cm in women), dyslipidemia, hypertension, and fasting hyperglycemia, is also tied to a number of risk factors for renal dysfunction.^6^ Severe obesity has been associated with increased measures of renal filtration, independent of clinical hypertension, or diabetes mellitus.^6^, ^7^, ^8^ In healthy young men, increasing BMI correlates with increasing creatinine clearance.^9^ However, the relationship between obesity and renal hyperfiltration has yet to be described in young healthy women in the absence of any pregnancy influence.

Of particular concern in young nulliparous women is the development of preeclampsia in their first pregnancy. Preeclampsia, typically characterized by hypertension and renal injury, is a significant contributor to both maternal and neonatal morbidity and mortality.^10^, ^11^, ^12^ Sensitive, cost‐effective, prepregnancy, and early‐pregnancy predictors remain elusive, and current recommendations for determining risk rely on patient history when deciding to initiate preeclampsia prophylaxis in nulliparous pregnant patients.^10^, ^13^ Obesity is a known risk factor, conferring a 1.6‐to‐3.3‐times increased likelihood of developing preeclampsia.^14^ The mechanism by which excess adiposity contributes to hypertensive disease of pregnancy is not well understood. Established chronic kidney disease, a major risk factor for preeclampsia, is associated with a fivefold increased risk,^13^, ^15^ but subclinical forms of renal dysfunction are not routinely assessed in patients.

The aim of this study is to determine the relationship between measures of obesity, renal filtration, and subclinical biochemical markers of metabolic syndrome in otherwise healthy, young, nulliparous women. Among a cohort of such women, it was hypothesized that a significant association exists between indices of obesity and biochemical markers of metabolic syndrome, including difference in renal vascular resistance.

## 2 METHODS

A secondary analysis of a single‐center cohort study was conducted, which included nulliparous women recruited as part of a prospective trial characterizing associations between prepregnancy and late‐pregnancy maternal physiology from November 2010 to June 2014.^16^ Eligible participants were between the ages of 18 to 42 years with self‐reported menstrual cycles of regular frequency and duration (from 26 to 35 days) who were planning pregnancy within the coming year. Excluded were women with preexisting diagnoses of hypertension, type 2 diabetes mellitus, autoimmune disease, or other major medical conditions.

Study participants were evaluated after a three‐day calorie, sodium, and potassium–controlled meal plan that was supplied to the patients as part of the protocol. Evaluations took place during the follicular phase of their menstrual cycle after an overnight fast. Participants were instructed to refrain from decongestants and nonsteroidal anti‐inflammatory medications for 48 hours prior to assessment, as well as caffeine and alcohol for 24 hours prior to evaluation.

Assessments were conducted in the University of Vermont Clinical Research Center. According to World Health Organization standards, overweight was classified as BMI 25.0‐29.9 and obesity was classified BMI ≥ 30.0. Adiposity was assessed via general electric lunar prodigy dual‐energy X‐ray absorptiometry (DEXA) scan (Software version 8.8, Madison, WI), which provided estimates of total and android body fat, as well as body surface area. Percent android body fat was calculated as android body fat divided by total body fat. As consensus standards have not been established for excess body fat, measures published by Gallagher et al that defined ≥ 33% body fat as overweight and ≥ 39% body fat as obese were applied.^17^ Blood pressure was evaluated with participants in the seated position following a period of rest using continuous noninvasive tonometric radial artery blood pressure monitoring. Doppler echocardiography, in left lateral supine position, was used to assess baseline cardiac output, and cardiac index was calculated by dividing by body surface area. Creatinine clearance was calculated based on 24‐hour outpatient urine collection. Serum cholesterol, triglycerides, high‐density lipoproteins, calculated low‐density lipoproteins, and homeostatic model assessment of insulin resistance were assessed after an overnight period of fasting.

Ultrasound indices of renal vascular resistance were assessed according to previously described methods.^18^, ^19^, ^20^ In brief, with subjects in supine position, a single expert operator (I.B.) assessed indices via an anterior approach using color Doppler ultrasound with a 3.5‐MHz curvilinear transabdominal transducer and a Vivid 7 general electric ultrasound unit (Milwaukee, WI). Examination took place with the lowest appropriate angle (0° to 60°). First, the main renal arteries were insonated with three to five reproducible waveforms taken from each kidney. Mean renal blood flow resistance index was calculated from these values via the Pourcelot equation ([peak systolic velocity – end‐diastolic velocity]/peak systolic velocity).^21^ Next, renal cortical blood flow resistance indices (CVRIs), a component of renal bed vascular resistance, were evaluated. Cortical arteries were likewise insonated with samples again obtained at cranial, middle, and caudal poles, before mean CVRI was calculated.

Descriptive statistical methods were used to characterize the study population. Means are reported with standard deviation. Pearson correlation and stepwise linear regression analyses were performed to evaluate and compare relationships between predictor variables and measures of renal vascular resistance. Regression analyses are reported as unadjusted. Scatterplots demonstrating relationships between measures of renal filtration, BMI, and percent android body fat were created using unadjusted values. Analyses were performed using SAS Version 9.1 statistical software (SAS Institute, Cary, NC).

The institutional review board of the University of Vermont approved this study. Informed consent was obtained and documented from each patient.

## 3 RESULTS

**Figure 1:**
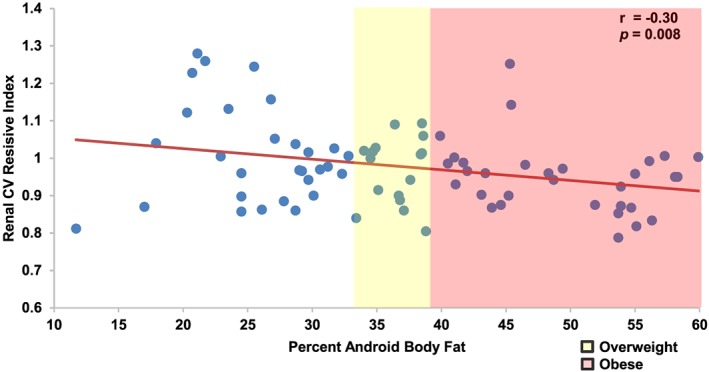
Renal cortical vessel resistive indices (CVRIs) are negatively associated with percent android body fat as measured via dual‐energy X‐ray absorptiometry scan in a cohort of young, healthy nulliparous women. Each subject (blue data point) is manifested with CVRI as it corresponds to percent android body fat. Linear fitting demonstrates a negative association. Standards proposed by Gallagher et al for overweight and obesity are overlaid in yellow and red, respectively, to demonstrate spectrum of android body fat among participants

Seventy‐nine healthy, nulligravid women consented and enrolled in the study. The cohort was young, with mean age 30.6 years. The group demonstrated demographic homogeneity, with 16% of participants being nonwhite and 4% identifying as Hispanic. Participants were relatively lean, with mean BMI 24.4 (Table 1). Only 17% of the cohort (n = 13) met criteria for obesity, whereas 15% (n = 12) were overweight. Two met criteria for severe obesity (3%). Mean android body fat was 38%, with 37% (n = 29) meeting criteria for lean adiposity (≤ 33%), whereas 43% (n = 34) met criteria for excess adiposity (≥ 39%). The cohort demonstrated evidence of cardiovascular fitness, with mean blood pressure 116/67 and a mean resting pulse of 66. Mean renal artery blood flow resistive index was 0.64, consistent with established normal indices.^20^ Normal range for CVRI has not been established; this cohort's mean was 0.98 ± 0.11. Mean HOMA‐IR (1.00 ± 0.50) demonstrated no evidence of clinically diagnosable insulin resistance. Cortical vessel resistive index was negatively associated with both percent android body fat (Figure 1) and BMI (r = ‐0.23, *p* = 0.047). Renal vascular resistance decreased with increasing measures of adiposity. Cortical vessel resistive index additionally was negatively associated with supine pulse (r = ‐0.44, *p* < 0.001). Other univariate predictors, including cardiac output, cardiac index, HOMA‐IR, and serum lipids, did not achieve significant associations with CVRI.

**Table 1:** Characteristics of subjects

	All	Normal	Overweight	Obese
	N = 79	N = 54	N = 12	N = 13
Age, y	30.6 ± 4.3	31.0 ± 4.4	30.0 ± 3.3	29.2 ± 4.4
Nonwhite race, No. (%)	13 (16.5)	10 (18.5)	2 (16.7)	1 (7.7)
Hispanic ethnicity, No. (%)	3 (3.8)	3 (5.6)	0 (0)	0 (0)
Height, cm	166.8 ± 7.2	166.7 ± 7.1	163.9 ± 8.2	163.8 ± 6.3
Weight, kg	67.0 ± 15.3	59.6 ±7.0	72.4 ± 7.3	93.2 ± 15.7
BMI, kg/m^2^	24.4 ± 5.4	21.4 ± 1.7	26.9 ± 1.5	34.5 ± 3.9
Android body fat, %	38.3 ± 12.1	32.4 ± 8.4	46.3 ± 9.5	55.4 ± 4.6
Resting supine pulse, bpm	66.2 ± 8.7	64.1 ± 8.5	69.6 ± 8.4	71.5 ± 6.7
Systolic blood pressure, mmHg	115.9 ± 9.1	115.4 ± 9.4	115.3 ± 9.2	118.7 ± 8.3
Diastolic blood pressure, mmHg	67.4 ± 6.0	66.2 ± 5.4	69.0 ± 5.1	70.9 ± 7.7
Mean arterial pressure, mmHg	86.9 ± 6.7	85.8 ± 5.6	88.9 ± 5.9	89.4 ± 9.4
Pulse pressure, mmHg	48.5 ± 8.4	49.2 ± 8.9	46.2 ± 8.3	47.8 ± 6.6
Cardiac output, L/min	4.5 ± 1.0	4.3 ± 0.8	4.5 ± 0.9	5.3 ± 1.2
Total peripheral resistance, MPa*s/m^3^	96.9 ± 20.9	99.8 ± 20.9	98.1 ± 21.3	84.1 ± 16.3
Renal artery blood flow resistive index	0.64 ± 0.05	0.65 ± 0.06	0.64 ± 0.04	0.60 ± 0.05
Renal cortical vessel blood flow resistive index	0.98 ± 0.11	1.00 ± 0.12	0.93 ± 0.08	0.93 ± 0.07
Creatinine clearance, mL/min	126.3 ± 31.8	121.2 ± 32.1	127.0 ± 34.2	146.1 ± 20.8
Homeostatic model assessment of insulin resistance	1.0 ± 0.5	0.9 ± 0.4	1.1 ± 0.5	1.5 ± 0.4
Data given as mean (SD) unless otherwise indicated.				

Abbreviations: BMI, body mass index; SD, standard deviation

**Figure 2:**
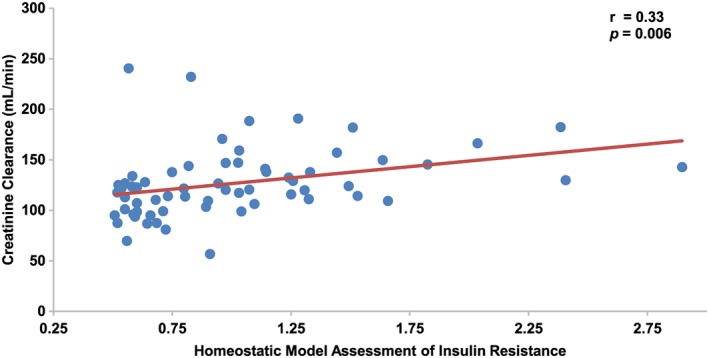
Creatinine clearance is positively associated with HOMA‐IR in a cohort of young, healthy nulliparous women. Each subject (blue data point) is manifested with creatinine clearance as it corresponds to body mass index. Linear fitting demonstrates a positive association

Creatinine clearance is positively associated with BMI (r = 0.29, *p* = 0.011). A positive association between creatinine clearance and percent android fat did not achieve statistical significance (r = 0.21, *p* = 0.07). HOMA‐IR (Figure 2) and cardiac output (r = 0.31, *p* = 0.005) each were significantly and positively associated with creatinine clearance. Other predictors, including supine pulse, cardiac index, and serum lipids, did not achieve significance in univariate analysis.

In regression modeling, CVRI was predicted by resting supine pulse (r^2^ = 0.22, *p* < 0.001) and cardiac index (r^2^ = 0.05, *p* = 0.045). Neither BMI nor percent android body fat remained independently predictive. Creatinine clearance was predicted by HOMA‐IR (r^2^ = 0.11, *p* = 0.008) and cardiac output (r^2^ = 0.06, *p* = 0.039). Again, neither BMI nor percent android body fat remained predictive.

## 4 DISCUSSION

In this cohort of young, healthy, nulliparous women, with predominantly normal BMI at evaluation, levels of obesity and adiposity are positively associated with increasing renal filtration. Additionally, subclinical insulin resistance was identified as an independent predictor of creatinine clearance. Renal filtration is demonstrated independently through both laboratory values and renal CVRI Doppler imaging. While increased measures of renal filtration have been documented in metabolic syndrome, obesity‐associated hypertension^2^, ^3^ and severe obesity,^7^, ^8^ this study demonstrates this correlation in healthy young nulliparous women without history of hypertension, significant obesity, or clinical insulin resistance. The findings suggest a spectrum of renal perfusion related to body fat and insulin resistance that may indicate early renal changes associated with preclinical metabolic syndrome.

In a cohort of women planning pregnancy in the coming year, these findings raise questions as to the implication of baseline renal function as it relates to adverse pregnancy outcomes. While long‐term metabolic and cardiovascular risks associated with preeclampsia have been well established,^22^, ^23^ mounting evidence suggests that preeclampsia may be an early manifestation of subclinical cardiovascular disease, as well as other elements of the metabolic syndrome including dyslipidemia and insulin resistance rather than an independent risk factor for the subsequent development of cardiovascular disease.^24^, ^25^, ^26^, ^27^, ^28^, ^29^, ^30^

Sensitive, cost‐effective prepregnancy and early‐pregnancy predictors of preeclampsia remain elusive,^10^ but the relationships described here might provide insights into mechanisms by which adiposity is associated with renal function and potentially predisposes to hypertensive disease in pregnancy. Identification of easily ascertained markers of preeclampsia risk may also identify candidates for antenatal prophylaxis, either with established options such as low‐dose aspirin^31^ or with emerging therapies.^32^ While current Bayesian approaches to screening for risk of preeclampsia factor in sonographic vascular bed assessment,^33^ they do not incorporate measures of adiposity, insulin resistance, or renal filtration into prediction models. From a clinical standpoint, creatinine clearance and BMI are routinely used measurements; DEXA and renal CVRI are not. However, clinically accessible measures of metabolic dysfunction, such as HOMA‐IR and waist circumference, could provide insights into those at risk of pregnancy‐associated renal dysfunction. Prospective associations should be evaluated.

This is a single‐institution prospective cohort study, which limits generalizability. This cohort is mostly white, and only 4% identify as Hispanic. Further research is warranted to study more ethnically and geographically diverse women in order to confirm associations between renal perfusion and measures of adiposity and insulin resistance.

Radiologic measures of small vessel renal filtration and adiposity have yet to be formalized. While consensus standards have not been established for excess body fat, Gallagher et al have defined ≥ 33% body fat as overweight and ≥ 39% body fat as obese.^16^, ^17^ Renal hyperfiltration is often defined only by laboratory assessment, but multimodal assessment offers further insight into the mechanism behind those values. Renal Doppler assessment is well described in the renal, lobar, and arcuate arteries,^18^, ^21^ and mean renal artery resistance index (0.64) is as would be expected. Assessment of the cortical vessels was based on the suspicion that small vessel vascular beds would show prepathologic changes in renal vascular resistance earlier than large ones and that resistance patterns in the arteries within the cortical region of the kidney would be most sensitive to small vessel disease. The location of these sonographically identified vessels correlates most closely with the inter‐lobular arteries. Confirmation of these findings in larger, more diverse populations would further validate these findings.

Strengths of this study include standardized, detailed physiologic evaluation with multimodal characterization of both obesity and renal filtration. This is the first report of such associations among healthy, nulliparous, reproductive‐aged women. Such women are generally at low risk of adverse health outcomes; however, in light of increasing rates of pregnancy‐related morbidity and mortality, these insights can play an important role identifying risks for renal dysfunction during an initial pregnancy.

## 5 CONCLUSION

Among a cohort of healthy, nulliparous women, renal vascular resistance decreases continuously with increasing obesity, whereas creatinine clearance and increasing insulin resistance is elevated with increasing BMI. This is consistent with a preclinical manifestation of renal hyperfiltration seen in metabolic syndrome, and may have important implications for a group of women planning reproduction in the near future as well as for their long‐term health.

## FUNDING

This study was supported by the National Institutes of Health under grant HL 71944.

## DISCLOSURE

The authors declare no conflict of interest.
